# A standardized quantitative method for detecting remnant alpha-Gal antigen in animal tissues or animal tissue-derived biomaterials and its application

**DOI:** 10.1038/s41598-018-32959-1

**Published:** 2018-10-18

**Authors:** Yan Lu, Anliang Shao, Yongqiang Shan, Hongni Zhao, Ming Leiguo, Yongjie Zhang, Yinxi Tang, Wei zhang, Yan Jin, Liming Xu

**Affiliations:** 1 National Institutes for Food and Drug Control, 102629 Beijing, China; 2 School of Medical Lab Science and life Science, Wenzhou Medical University, 325035 Wenzhou, China; 3 Subei People’s Hospital of Jiangsu Province, 225001 Jiangsu, China; 4 Research and Development Center for Tissue Engineering, Fourth Military Medical University, 710032 Xi’an, China; 5 National Engineering Laboratory for Regenerative Medical Implant Devices, Guanhao Biotech, Co., LTD, 510530 Guangzhou, China

## Abstract

Alpha-Gal (Gal) epitopes present in animal tissues are known to be the key xenoantigens that elicit xenorejection. However, a standardized method to determine Gal epitope in animal tissue-derived biomaterials does not exist. Herein, a standardized method for quantitative detection of Gal antigen was established based on an ELISA inhibition assay with Gal antibody. In this method, the key optimized experimental conditions were: (1) Gal-antigen positive and negative reference materials were developed, and used as positive and negative control in the test system, respectively; (2) A mixture of artificial Gal-BSA antigen plus Gal-negative matrix was used as the calibration standard sample, making it has similar composition with test sample; and (3) The lysis buffer was combined with the homogenate to expose the Gal antigen as much as possible. The results from validation and application experiments showed that the standardized method had good reproducibility (RSD = 12.48%), and the lower detection limit (LDL) is ~7.1 × 10^11^ Gal epitopes/reaction. This method has been further developed into a detection Kit (Meitan 70101, China), and it has been developed as a standard method for detecting remnant immunogen of animal tissue derived medical devices, and as the industry standard has been released in China. (YY/T 1561–2017).

## Introduction

A shortage of organ donors has led humans to explore the field of xenotransplantation by using the tissue or organ of animals. Currently, animal tissue-derived biomaterials have been widely used as regenerative medical products. Gal antigen (α1,3 Gal epitopes) is the major xenoantigen that causes hyperacute rejection (HAR) in xenotransplantation (e.g. pig-to-human)^1–3^. The remnant Gal antigen in animal tissue-derived biomaterials is considered to play an important role in eliciting immunological responses in the human body, which further affects the tissue remodeling outcomes^4^. All non-primate mammals (e.g., mice, rats, rabbits, dogs, pigs, etc.) as well as prosimians (lemurs) and New World monkeys (monkeys of South America) have been reported to produce ~10^5^–10^7^ α-gal epitopes/cell^5^. In contrast, Old World monkeys (monkeys of Asia and Africa), apes and humans do not produce the α-gal epitope because they lack the glycosylation enzyme α1,3galactosyltransferase (α1,3GT) synthesizing this epitope^1^. However, continuous antigenic stimulation by the gastrointestinal flora results in production of ~1–3% of natural anti-Gal Ab in these primates^6–8^. Therefore, when xenotransplants or animal tissue-derived biomaterials containing Gal antigens are transplanted into the human body, they likely contribute to HAR or chronic immune rejection, and further delay tissue repair and re-construction^9,10^.

In order to overcome the hyperacute immune rejection of the xenotransplanted tissues or organs, and chronic immune rejection of animal tissue-derived biomaterials, methods like decellularization and/or chemical cross-linking that remove or mask the antigen epitopes are utilized. On the other hand, the production of homozygous pigs with a disruption in *GGTA1* gene^11–13^ to make Gal antigen unexpressed clearly represented a critical step toward the clinical reality of xenotransplantation. Therefore, assessing Gal antigen residues in decellular animal tissue-derived biomaterials is important to predict potential immunological risk.

At present, there are no standardized methods to accurately quantify the expression or remnant Gal antigen in animal tissues or animal tissue-derived biomaterials. Xenoreactive antigens are often studied by using the lectin *Griffonia simplicifolian* 1 isolectin B4 (IB4), which shows high affinity to galactose. Using radiolabeled IB4 staining to detect Gal on the animal cells, Galili *et al*. demonstrated that the radioactivity was detected in different cells of Gal antigen epitopes, e.g. 1.2 × 10^6^ per myeloma cell^1^. Thall A *et al*. established a sensitive solid-phase radioimmunoassay (ELISA inhibition assay) based on the binding of anti-Gal to various cells, which also correlated with the binding of lectin Bandeiraea (*Griffonia*) *simplicifolia* IB4^14^. IB4 was also used to analyze Gal antigen expression in *GGTA1* knockout animals or cells, and the results showed that fetal fibroblasts derived from GGTA1 KO pig or GGTA1 KO mice were Gal α1,3 Gal negative^11–13,15,16^. However, the specificity of IB4 binding to different sugars in Gal antigen is varied. Several studies have shown that IB4 lectin is insufficient for the detection of relatively small number of Gal epitopes because of low binding affinity of the monomeric interaction of the lectin molecule with more than 1 of the 4 combining sites^17^. Kirkeby *et al*. found that the length of the sugar chains influenced the lectin-carbohydrate interaction, and not only the terminal, but also the subterminal sugar affected lectin binding^18^. Galili *et al*. developed a highly specific antibody for alpha-Gal — monoclonal anti-Gal IgM also called M86 — to establish an ELISA inhibition assay that quantitatively detects the Gal epitopes. Using this ELISA method, they quantified the Gal epitopes of tissues or organs from the standard curve of Gal epitope expression in the myeloma cells or rabbit red blood cells^17^. According to previous studies, myeloma cells expressed about 1.2 × 10^6^ Gal epitopes per cell when detected by IB4^1^, and the sensitivity of rabbit red blood cells was found to be 2-fold compared to myeloma cells, suggesting that the Gal epitope expressed on rabbit red blood cells was about 2 × 10^6,17^. Based on these data, rabbit red blood cells along with standard materials have been utilized to produce calibration curves for detecting Gal antigen in animal tissues in the test system of ELISA inhibition assay with monoclonal M86 antibody^17,19^. These measurements allow for relative quantitative determination of Gal epitopes, but do not provide an accurate calculation of the number of Gal epitopes per cell or per biomaterials (e.g. per mg).

Based on the studies described above, several questions need to be considered: (1) Gal antigen epitope expression of myeloma cells or rabbit red blood cells used for establishing the standard curve is inaccurate and difficult to be traced; (2) It is unknown whether there are differences in the Gal antigen epitope expression between different generations of myeloma cells. Also, it is unclear whether the Gal antigen expression differs between various species and age of rabbit; (3) Test samples (animal tissues or animal tissue-derived biomaterials) are usually in a matrix containing Gal antigen, whereas standard samples are mouse myeloma cells or rabbit red blood cells, thereby producing different Gal antigen-antibody reaction systems. Using cells as a reference to detect the Gal antigen in biomaterials is therefore not sensible; (4) If Gal antigen-positive and Gal antigen-negative reference material were not used in the test system, the method was unable to recognize false-positive and false-negative results, and were therefore excluded; (5) Mechanical homogenization^17^ may not result in the farthest exposure of the antigen from animal tissues or animal tissue-derived biomaterials. Moreover, incomplete inactivation of the enzyme following next step in the enzyme (e.g. papain) digestion method^20^ may result in a false-positive. Due to these uncertainties, the results obtained from different laboratories are not comparable.

In order to solve the above problems, we used commercially available artificial Gal-BSA antigen as a standard material to redefine the Gal epitopes expressed in mouse myeloma cells or rabbit red blood cells with M86 ELISA inhibition assay. We then used mouse myeloma cells or rabbit red blood cells as standard material to establish refined M86 ELISA inhibition assay for the detection of Gal epitopes in animal tissues or animal tissue-derived decellular biomaterials^20,21^. Also, Gal antigen-positive and Gal antigen-negative reference materials were used to monitor the sensitivity and specificity of test system^20,21^. However, the results to be detected do not represent the actual results of animal tissues or animal tissue derived biomaterials when using mouse myeloma cells or rabbit red blood cells as standard samples due to the Gal antigen-antibody reaction system were quite different.

In the present study, we established a more refined and standardized method for quantitative determination of remnant Gal antigen in animal tissues or animal tissue-derived biomaterials based on previously reported ELISA inhibition assay using M86 antibody^19–21^ with several modifications. Briefly, a mixture of Gal antigen-negative reference material/Gal-BSA (commercially available) artificial Gal antigen, which was similar in composition with the test sample (animal tissues or animal tissue-derived biomaterials), was used as the reference sample for generating a calibration curve. To fully expose the antigen from solid material as much as possible the test sample was prepared using mechanical homogenization plus lysis buffer. Gal antigen-positive and Gal antigen-negative reference standard materials were developed, and introduced in the test system to monitor sensitivity and specificity. Using this standardized method, we quantitatively determined the Gal antigens present in animal tissues, GGTA1 KO pig cells as well as remnant Gal antigens in animal tissue-derived biomaterials (biological dural graft derived from bovine pericardial membrane). This standardized method has been published in China as an industry standard for medical devices.

## Results

### Identification of Gal antigen content and homogeneity in the reference materials

The Gal antigen was undetectable based on the OD values of Gal antigen-negative reference material (1.24 ± 0.23), and no significant differences were found when compared with the OD values of 100% reaction control (non-inhibitory control) (1.16 ± 0.18), indicating that the Gal antigen-negative reference material did not contain Gal antigen (Table 1).

**Table 1:** The OD value of Gal antigen-negative reference material compared to 100% control (n = 9).

	100% control (M86 + lysis buffer)	Gal antigen-negative reference material
OD	0.93	1.12
	1.30	1.64
	1.33	0.92
	1.10	1.14
	1.36	1.17
	1.07	1.10
	1.37	1.30
	0.99	1.55
	1.00	1.20
Mean	1.16	1.24
SD	0.18	0.23
CV (%)	15.52	18.55

To identify the Gal antigen content and verify the homogeneity of the Gal antigen-positive reference material, Gal-BSA/Gal antigen-negative reference material mixture (Gal-BSA mixture) was used as standard materials to generate a calibration curve, and the Gal content was determined by M86 ELISA inhibition assay as demonstrated in this study. The results showed that the Gal antigen content of the Gal antigen-positive reference material was (6.66 ± 0.83) × 10^14^/mg with 12.48% of relative standard deviation (RSD, n = 9), which was calculated based on the calibration curve of Gal-BSA mixture (Table 2). The coefficient of variation (CV) in the results was less than 15%, indicative of good homogeneity.

**Table 2:** The Gal antigen content and homogeneity verification of Gal antigen-positive reference material (n = 9).

Test Group	The number of Gal antigens (×10^14^/mg)
Test 1	5.78
Test 2	6.88
Test 3	6.81
Test 4	5.45
Test 5	6.65
Test 6	6.83
Test 7	6.65
Test 8	6.44
Test 9	8.43
Mean	6.66
SD	0.83
RSD (%)	12.48

### Stability verification of Gal antigen-positive reference materials

Stability of Gal antigen-positive reference materials was verified by storing the samples under the following conditions: Store at −20 °C for 1, 6, and 12 months; freezing/thawing for 1, 3, and 5 times; store at 4 °C for 3, 7, 14 and 30 days; store at 25 °C for 3, 7 and 14 days; and store at 37 °C for 1, 3 and 7 days. As shown in Table 3, only samples stored at 37 °C for 3 and 7 days caused a significant decrease in Gal antigen content [(4.34 ± 0.81) × 10^14^/mg and (4.67 ± 0.69) × 10^14^/mg, respectively] compared to the identified value [(6.66 ± 0.83) × 10^14^/mg] of Gal antigen-positive reference material (p < 0.05). The results suggested that the samples were stable in all of these conditions, except for those stored at 37 °C. Considering the quality assurance, we recommend storing the reference materials at −20 °C, and freezing/thawing not more than 5 times.

**Table 3:** Verification of stability of Gal antigen-positive reference materials.

Storage conditions of the samples^#^	Gal epitopes (×10^14^/mg)
# of months stored at −20 °C	
1	6.06 ± 0.20
6	6.71 ± 0.10
12	6.97 ± 0.20
# of times of freezing/thawing	
1	6.49 ± 0.62
3	6.73 ± 0.15
5	6.48 ± 0.23
# of days stored at 4 °C	
3	6.40 ± 0.49
7	6.31 ± 0.40
14	6.16 ± 0.49
30	5.87 ± 1.02
# of days stored at 25 °C	
3	7.49 ± 0.72
7	6.70 ± 1.83
14	7.10 ± 1.19
# of days stored at 37 °C	
1	6.24 ± 1.31
3	4.34 ± 0.81^*^
7	4.67 ± 0.69^*^

^*^p < 0.05 compared to the identified value [(6.66 ± 0.83) × 10^14^/mg] of Gal antigen-positive reference material (stored at −20 °C); ^**#**^n = 3.

### Sensitivity and specificity of the test system

**Figure 1:**
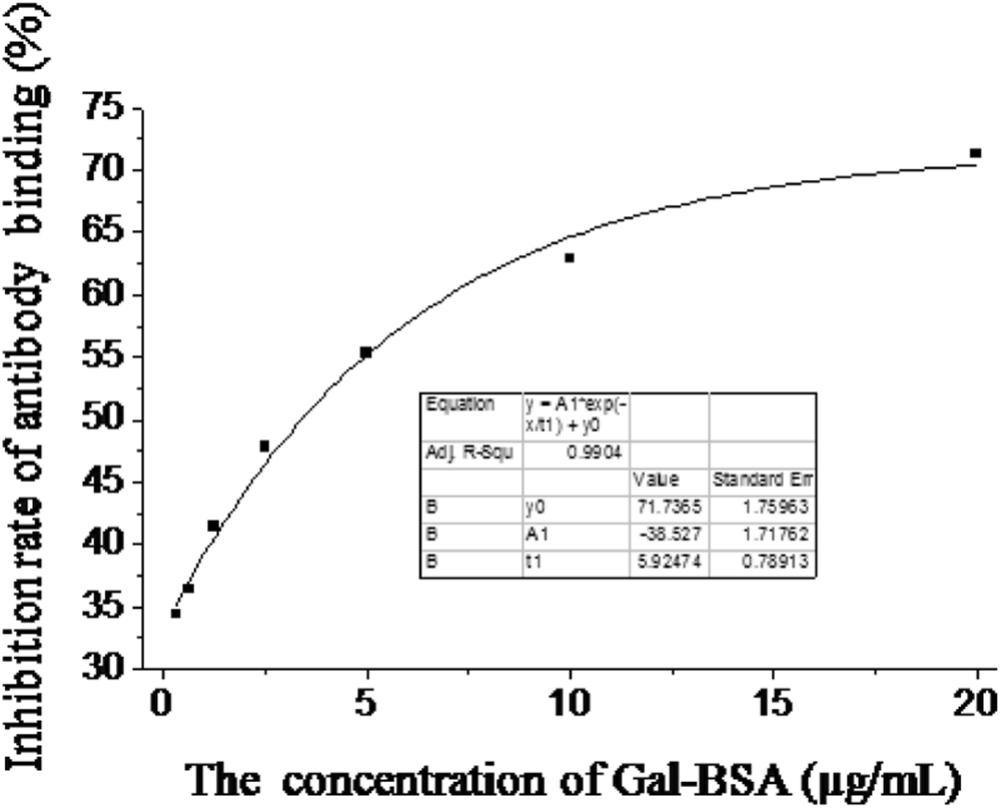
The standard curve of Gal-BSA mixture. The X-axis is concentration of Gal-BSA (µg/mL) and Y-axis is inhibition rate of antibody binding (%). Indication: Origin 8 graphics program was used to create the artwork, and save them as tiff.

Gal antigen-positive reference material was introduced into the test system in order to monitor sensitivity of the experimental method. Gal antigen-positive reference material was made from porcine tissue that contained Gal antigens as much as (6.66 ± 0.83) × 10^14^/mg freeze-dried homogenate. The results showed that the value of Gal antigens present in Gal antigen-positive reference material was (6.76 ± 0.46) × 10^14^/mg freeze-dried homogenate (n = 3). Moreover, the CV was less than 15% compared to its defined value, and the lower detection limit (LDL) was about 39 ng to 78 ng relative to Gal-BSA (which is equal to 7.1 × 10^11^ Gal epitopes/reaction calculated from the formula derived from Fig. 1, suggesting that the test system has enough sensitivity.

To monitor the specificity of this method, a Gal antigen-negative reference material was introduced into the test system. Gal antigen-negative reference material was made from human tissue, which theoretically does not contain Gal antigen, and was further verified in this study during the development of reference standard material. The data showed that the OD value of Gal antigen-negative reference material (1.26 ± 0.12) was not significantly different (*P* > 0.05) compared to the OD value of 100% reaction control (non-inhibitory control, 1.15 ± 0.02), suggesting that the sample preparation (homogenization plus lysate with lysis buffer) did not generate false-positive results, and therefore has good specificity.

### Test method validation for repeatability

In order to investigate the repeatability of the established test method, Gal antigen determination of Gal antigen-positive reference material was repeated 4 times (inter-day and intra-day), and the CV of 50% inhibition rate (IC_50_) of M86 binding between each time calculated. As shown in Table 4, the inter-day results for the IC_50_ (µg/mL) of M86 binding of Gal antigen-positive reference material was 6.49 ± 0.52, the CV was 8.07%, whereas the intra-day results had an IC_50_ (µg/mL) 5.37 ± 0.66, with the CV of 12.28%. The total (inter-day and intra-day results) IC_50_ (µg/mL) of Gal antigen-positive reference material was 5.93 ± 0.81, the CV was 13.73%. These results indicated that the test method has good repeatability.

**Table 4:** Test method validation for repeatability (n = 3).

	IC50 of Gal antigen-positive reference material related to Gal-BSA (µg/mL)						
	n1	n2	n3	n4	mean	SD	CV (%)
inter-day	6.65	6.62	5.73	6.94	6.49	0.52	8.07
intra-day	6.08	5.71	5.07	4.60	5.37	0.66	12.28
Total	6.65	6.62	5.73	6.94	5.93	0.81	13.73
	6.08	5.71	5.07	4.60			

### Gal antigen expression in test samples

By using the standardized method demonstrated above, Gal antigen expressed in animal tissues (porcine, bovine), animal tissue derived biomaterials (biological dural graft made from pericardial membrane of bovine), and wild type (WT) porcine fibroblast cells and GGTA1 knockout (KO) porcine fibroblast cells were determined. The results are shown in Tables 5 and 6.

**Table 5:** Gal antigen expression in the tissues and tissue-derived biomaterials (n = 3).

Tissue/biomaterial	Gal epitopes/mg	Antigen reduction (%)
Porcine kidney tissue (Freeze-dried)	(6.32 ± 1.01) ×10^17^	
Porcine dermal tissue (Freeze-dried)	(5.92 ± 1.63) ×10^15^	
Porcine cornea tissue (wet)	(1.54 ± 0.55) ×10^13^	
Bovine pericardial membrane (wet)	(2.49 ± 0.50) ×10^15^	
Biological dural graft (wet)	(1.6 ± 0.02) ×10^13^	99.36%*

^*^p < 0.05, Gal antigen was significantly decreased compared to fresh bovine pericardial membrane.

**Table 6:** Gal antigen expression in porcine fibroblasts.

cell	Related Gal-BSA (×10^−7^ µg/Cell)	Epitopes/Cell	Antigen reduction (%)
GGTA1 KO porcine fibroblasts-1	0.57	1.03 × 10^7^	99.15%^*^
GGTA1 KO porcine fibroblasts-2	3.73	6.42 × 10^7^	94.73%^*^
WT porcine fibroblasts	67.43	1.22 × 10^9^	

*p < 0.05, Gal antigen was significantly decreased compared to WT porcine fibroblasts.

Porcine and bovine tissues or organs are most commonly used in the field of xeno-transplantation and biological materials production, therefore detecting the Gal epitope residues in the tissues or biological material products is very useful for assessing or predicting the immunological risks in its intended clinical use. The results demonstrated that porcine kidney tissue (freeze-dried), porcine dermal tissue (freeze-dried), fresh porcine cornea (wet), and fresh bovine pericardial membrane (wet) expressed the Gal epitopes as much as (6.32 ± 1.01) × 10^17^/mg tissue, (5.92 ± 11.63) × 10^15^/mg tissue, (1.54 ± 0.55) × 10^13^/mg tissue, and (2.49 ± 5.04) × 10^15^/mg tissue, respectively (Table 5). Compared to fresh bovine pericardial membrane, the product of biological dural graft (wet) made from fresh bovine pericardial membrane with decellular procession showed significantly decreased remnant Gal antigen to as much as 99.36%, with a value of (1.6 ± 0.02) × 10^13^/mg tissue.

The production of homozygous pigs with a disruption in *GGTA1* gene^11–13,22^ expecting to make Gal antigen unexpressed has been widely studied for the clinical reality of xeno-transplantation. But, whether *GGTA1* gene knock out yields completely absent Gal epitopes has different conclusions currently, which may be due to different detection methods used in these studies. In this study, Gal epitope expression in the fibroblasts derived from *GGTA1* KO mini-pig was determined, and the result showed that Gal epitope expression significantly decreased to as much as 99.15% and 94.73% compared to Wild-type mini-pig fibroblasts (Table 6), but was still expressed (1.03 × 10^7^ epitopes/cell and 6.42 × 10^7^ epitopes/cell).

## Discussion

Gal antigen is the main target antigen for immune rejection in xeno-transplantation^23^. Detecting the Gal antigen or its remaining in animal tissues or animal tissue-derived biomaterials can therefore be used to evaluate the reduction of xeno-immunogens in those biomaterials, and to forecast its risks of immunogenicity. In this study, we developed a Gal antigen-positive reference material and a Gal antigen-negative reference material, and the results from the verification experiments have been used for registered application of national reference material. These reference materials are currently approved as national reference materials in China.

We then established a standardized quantitative method for determining the remnant Gal-antigen in animal tissues or animal tissue-derived biomaterials. In this standardized method, several key steps were modified based on previously reported ELISA inhibition assay with monoclonal M86 antibody^17,19–21^. Firstly, to monitor the sensitivity and specificity of the test system, we set the positive and negative control with Gal antigen-positive and Gal antigen-negative reference material, respectively. For a standardized method, using positive and negative reference materials are very useful to monitor the specificity and sensitivity of the test system. Without using positive and negative reference material in the detection system, the results obtained from different laboratories are difficult to compare, and it also lacks credibility.

Second, we used artificial Gal-BSA antigen plus Gal-negative matrix (Gal antigen-negative reference material) to make a mixture which has similar composition with the test sample (animal tissue or animal tissue derived biomaterial), and used it as a calibration standard sample. It is very important that the antigen-antibody reaction between standard and test samples have similar efficiencies under the same experimental conditions. Various studies have reported Gal antigen detection, but the results between different laboratories are not comparable. For example, controversial conclusions still exists on whether *GGTA1* KO pigs or mice yield completely unexpressed Gal antigen, based on current results reported by different groups, and this may be because different methods for Gal antigen detection were used in different laboratories. Previous studies on the analysis of *GGTA1* KO pig fetal fibroblasts with IB4 lectin showed that the cells were alpha1,3 Gal negative^11–13,22^, and was in accordance with the data obtained for *GGTA1* KO mice^15,16^. However, the results obtained by using anti-Gal mAbs showed that a wide range of tissues in *GGTA1* KO mice were Gal antigen positive^23^. Sharma *et al*. showed that cells from *GGTA1* KO pig lines also express low levels of alpha1,3 Gal when detected with anti-Gal mAbs^24^. In this study, we also detected the Gal epitope expression in the ear fibroblasts derived from the *GGTA1* KO mini-pig, which are reported to have unexpressed Gal antigen^22^, and the result from our detection showed that Gal epitope expression was significantly decreased compared to wild-type mini-pig fibroblasts, but still expressed (1.03 × 10^7^ epitopes/cell and 6.42 × 10^7^ epitopes/cell). Inconsistent results were obtained even when the anti-Gal mAb (M86) was used for the detection of Gal antigen in animal tissues between different laboratories. For example, Galili *et al*. reported that Gal antigens expressed in porcine kidney tissue was 1 × 10^12^/mg^25^, whereas our results showed 1.02 × 10^17^/mg of Gal antigens in the porcine kidney tissue, which was calibrated by SP2/0 standard curve from our previous study^21^, and (6.32 ± 1.01) × 10^17^/mg calibrated by Gal-BSA mixture standard curve in this study. Both the studies used M86 antibody in the detection system however, Galili *et al*. used SP2/0 cells or rabbit red blood cells as standard calibration and identified their Gal epitope expression by IB4 lectin staining as much as about 1 × 10^6^ per cell (the traceable data was 1.2 × 10^6^/cell) in SP/0 cells^1,17^ and 2 × 10^6^ per cell in rabbit red blood cells^17^. We re-identified Gal epitope expression in SP2/0 cells and rabbit red blood cells by using artificial Gal-BSA as calibration standard, the values for which were 6.19 × 10^8^ per cell^21^ and 2.73 × 10^7^ per cell^20^, respectively. Different calibration standards used in the detection system cause significant differences in the results.

Third, sample preparation is also a key step. Many studies prepare tissue samples by homogenization or papain digestion, in order to expose the Gal antigens. However, diverse homogenizers are available in the market, which make it difficult to assure full exposure of the Gal antigens. Papain digestion ensures that the Gal antigen is fully exposed however, after the digestion it is difficult to assure 100% papain enzyme inactivation by simply heating the digested solution at 100 °C for a few minutes. Incomplete inactivation of papain enzyme may affect (destroy) the Gal antigen and antibody in the reaction solution, and decrease down-step Gal antigen-antibody reaction efficiency, thereby generating false-positive results. We encountered this problem in our preliminary experiments that caused the Gal antigen was positive in Gal antigen-negative reference material however, using H_2_O_2_ to inactivate the papain enzyme, we verified the negative results^21^. Tissue or cell lysis buffer containing protein protection reagents is often used for lysing the tissue and cells in many biological studies^26–29^. We combined lysis buffer with the homogenate to expose the Gal antigen containing in the tissues or tissue-derived biomaterials as much as possible. In our preliminary experiments, we confirmed that the lysis buffer did not affect the reaction efficiency of down-step antigen-M86 antibody reaction by comparing the response value with solid-phase antigen between M86/lysis buffer and M86/PBS solution (data not shown).

Lastly, the M86 ELISA inhibition method reported in the previous studies used 50% inhibition rate of M86 binding to obtain relative quantitative results. However, de-cellular animal tissue derived biomaterials do not usually contain high levels of remnant Gal antigens, therefore, it may not reach 50% inhibition rate of M86 binding in the test system. In the present study, a single concentration was detected by quantitative calibration curve generated from Gal-BSA mixture. The lowest limit of determination in every experiment was found by increasing lower dilutions in such a way the samples are undetectable, so the upper one concentration of that is the lowest detectable limit (LDL). The LDL in our test system was 39 ng Gal-BSA (about 7.1 × 10^11^ Gal epitopes/reaction can be detected).

This standardized method was verified by different laboratories according to the standardization principles of medical device industry in China. Variations in intra- (Table 2) or inter-laboratory (data not shown) were less than 20%, indicating a high reproducibility of the method. As an industry standard using for animal tissue derived medical device, it has been approved by Sub-technical Committee of Tissue Engineering Medical Devices/ Technical Committee for Surgical Implants and Orthopedic Instruments/ Standardization Administration of China (SAC/TC110/SC3), and this industry standard has been released in 2017 (YY/T 1561–2017, Tissue engineering medical device products - remnant α-Gal antigen determination in scaffold materials utilizing animal tissues and their derivatives. China industry standard released in 2017).

## Conclusions

We established a standardized method with high specificity and sensitivity for quantitative determination of Gal antigen. In this method, several experimental conditions were refined based on the literature and our previous studies. The key modifications were: (1) Gal antigen-positive and Gal antigen-negative reference materials were developed, and used as positive and negative control, respectively, in test system; (2) A mixture containing artificial Gal-BSA antigen plus Gal-negative matrix was used as a calibration standard sample, making it had similar composition with test sample; (3) Combining lysis buffer with the homogenate to expose the Gal antigen containing in the tissues or tissue-derived biomaterials as much as possible. The standardized method has good reproducibility and can determine as lower as about 7.1 × 10^11^ Gal epitopes/reaction in animal tissue derived biomaterials. This method has been further developed into a detection Kit (Meitan 70101, China)^29^, and it has been developed as a standard method for the detection of remnant immunogen in animal tissue derived medical devices, and has been released in China as an industry standard (YY/T 1561–2017).

## Materials and Methods

### Reagents

Gal α 1-3Gal-BSA (Gal-BSA, Dextra Laboratories Ltd, NGP0203), α-Gal epitope (Galα1-3 Galβ1-4 GlcNAc-R) antibody, M86 mAb (Enzo Life Sciences, ALX-801-090-1), goat anti-mouse IgM-HRP (Santa Cruz Biotechnology, sc-2064), Human Serum Albumin (HSA) (Sigma, A8230-1), TMB (Beijing Branch of Biotechnology LTD, SE1005), RIPA lysis buffer (Beyotime, P0013B), PMSF (Beyotime, ST506), Tween-20 (Solarbio, No. D042).

### Experimental instruments

Homogenizer (Benchmark, D1000-E), microplate reader (Molecular Devices, Spectramax M5), standing-temperature cultivator (Taicang Haocheng, HZQ-X100), centrifuge (Sigma, 3k15), Benchtop (bgwaPeec, CJT-90) Inverted Microscope (Olympus, CKX41SF), Vortex suspension device (US SI, Genie 2), High precision scales (Shanghai Mingqiao, FA1104N), standing-temperature water bath (BEIJING ERA BEILI, HW-60), Costar 96 well plate (Corning Costar, 2592).

### Experimental samples

Freeze-dried porcine tissues (Kidney, Dermal) were a gift from Professor Yan Jin (Fourth Military Medical University), whereas wild type (WT) and GGTA1 knockout (KO) porcine fibroblast cells were gifted by Professor Liangxue Lai (Key Laboratory of Regenerative Biology, Chinese Academy of Science, and Guangdong Province Key Laboratory of Stem Cells and Regenerative Medicine, South China Institute for Stem Cell Biology and Regenerative Medicine, Guangzhou Institutes of Biomedicine and Health). Fresh porcine cornea, bovine pericardial membrane, and biological dural graft derived from pericardial membrane were provided by Guanhao Biotech (Batch numbers: 150301-1, 150301-3). Gal antigen-positive (380001) and Gal antigen-negative reference materials (380002) developed and demonstrated in this study were provided by the National Institutes for Food and Drug Control, China.

### Experimental sample preparation

#### Standard sample

Gal-BSA (commercially available) was used as a standard material, which possesses ~1.82 × 10^20^/g Gal epitopes^20,21^. To ensure similar composition between the standard materials and test samples, 2–10 mg of freeze dried Gal antigen-negative reference material was combined with Gal-BSA as standard sample (Gal-BSA mixture). Briefly, lysate for Gal antigen-negative reference material was prepared in lysis buffer containing 1% protease inhibitor PMSF, and incubated for 1–3 h at room temperature. Gal-BSA (500 μg/mL, stored at −20 °C) was then diluted at least five different concentrations (e.g. 20, 10, 5, 2.5, 1.25, 0.625, 0.313, 0.156, 0.078, and 0.039 μg/mL) using the lysate of Gal antigen-negative reference material. However, Gal-BSA was diluted in the cell culture medium or lysis buffer when determining Gal antigen in the test sample of cells in order to keep test system similar to the cell sample. To find the lowest limit of determination in every experiment, increasing lower dilutions as lower as finding-out not detectable sample is necessary, so the upper one concentration of that not detected sample is the LDL.

#### Test sample

For solid samples, the lysate was prepared by homogenizing them in certain amount of lysis buffer (e.g. 2–10 mg/1–2 mL) containing 1% protease inhibitor PMSF, and incubating at room temperature for 1–3 h, and making sure that there were no obvious solid matters and the Gal antigen was exposed as much as possible. The lysates for liquid or gel samples were directly prepared in the lysis buffer, whereas to obtain the cell lysates, cells were first diluted in cell culture medium or directly lysed in the lysis buffer after washing in PBS.

#### Reference material

The first, reference material was developed according to the “reference material management method” delivered by National Metrology Bureau, China (July 10, 1987) and the requirement of “National pharmaceutical and medical device reference material management system”. Gal epitopes are known to be expressed in all of the non-primate animals, but not in Old World monkeys, apes, and humans^1,5^. Therefore, porcine and human tissues were selected as raw materials to develop Gal antigen-positive and Gal antigen-negative reference materials. Based on the principles of selecting raw materials (easy to process, easily homogeneous, easy to obtain starting materials), porcine liver tissue and human placenta tissue were selected as starting materials for reference sample development. All experiments related to the use of human tissue samples were performed in accordance with relevant guidelines and regulations (National Health and Family Planning Commission of the People’s Republic of China), and informed consent was obtained from all subjects.

To ensure that the sample has good homogeneous properties and is stable, we chose the tissue homogenate (at 4 °C) and freeze-dried dosage form as the production process, and the samples were stored at −20 °C. The processing of the standard reference materials was completed with the cooperation from the laboratory of Fourth Military Medical University and Chengdu Qingshan Li Kang Pharmaceutical Co. Ltd. The ethical review of human tissue acquisition was approved by local ethics committee, and the document (informed consent was obtained from all subjects) was kept in Chengdu Qingshan Li Kang Pharmaceutical Co. Ltd. The value of Gal antigen content was used as an identified value of reference material, and the homogeneity and stability were verified by three laboratories.

To monitor the test sensitivity and specificity, Gal antigen-positive and Gal antigen-negative reference materials were used in the experimental system. For the reference material samples, the lysates were prepared in the lysis buffer, similar to the test sample preparation.

#### Solid Gal antigen coating

Preliminary experiments were performed to refine the coating conditions at several concentrations of Gal-BSA (2.5, 2.0, 1.0, 0.5, 0.25, 0.1, and 0.05 μg/mL) in order to identify the concentration at which the variations in the test results were minimum (data not shown). The concentration of 2 μg/mL of Gal-BSA was found can generate minimum variations, and was chosen to coat the 96-well plates (100 μL/well). The coated plates were incubated for 2 h at room temperature with gently shaking (100 rpm), and further incubated at 4 °C overnight. After 3 washes with 0.05% tween-20/PBS, the plates were incubated in 200 μL/well of 1% HSA/PBS for 2 hrs at 37 °C with gently shaking (100 rpm) to block the non-specific binding sites. Following 3 washes, the Gal-BSA coated 96-well plates were slightly dried by discarding the wash solution and tapping on paper towels, sealed by a sealing membrane, and stored at 4 °C until further use. The long-time storage may reduce the antibody-antigen reaction efficiency, and therefore we recommend using the coated plates within one week.

### ELISA inhibition assay

A mouse monoclonal anti-Gal IgM antibody (M86) was used to detect Gal-antigens. Briefly, 100 μL lysate of standard samples (Gal-BSA mixture), reference material samples and test samples were incubated with 100 μL of M86 (1:100) at room temperature for 2 h with gently shaking (100 rpm), and left at 4 °C overnight. All experimental samples were set as 3 parallel samples. During incubation, the M86 antibody (final concentration 1:200) binds with the Gal epitopes present in the experimental samples. After incubation, the samples were centrifuged at 14,000 g for 30 min at 4 °C to remove the matrix and Gal antigen-bound antibody molecules.

The activity of M86 remaining in the supernatant was determined by ELISA with Gal-BSA as a solid-phase antigen and horseradish peroxidase-conjugated goat anti-mouse IgM antibody as a secondary antibody. Briefly, 100 μL of supernatant containing M86 was loaded in a 96-well plate (coated with Gal-BSA overnight), and incubated for 2 h at 37 °C with gently shaking (100 rpm). After three washing, the secondary goat anti-mouse IgM-HRP antibody diluted in PBS (1:1,000) was loaded and incubated for 1 h at 37 °C with gently shaking (100 rpm). Following three washes, 100 μL of horseradish peroxidase substrate buffer tetramethylbenzidine (TMB) was added to each well, and the plates were incubated in the dark for 15 min at room temperature. Finally, 50 μL of 10% H_2_SO_4_ was added to each well to stop the reaction, and the absorbance was read at 450 nm within 3–5 min using a microplate reader.

Because the mixture of Gal antigen/M86 antibody formed in the first Gal antigen – M86 antibody reaction was removed before the ELISA, the interaction resulted in “inhibition” of the subsequent binding of remaining M86 to the solid phase Gal-BSA. In this experiment, the well containing M86 antibody (1:200, diluted in lysis buffer or lysate of Gal-negative reference material, the composition similar to the test sample) alone was set as 100% reaction control (non-inhibitory control), and the well containing lysis buffer alone was set as blank.

### Calculation and analysis of the results

At first, the M86 inhibition rate of all experimental samples was calculated using the formula shown below:

1 $$\frac{(a-b)-(c-d)}{(a-b)}\times 100 \% $$

where*a* = OD for 100% reaction control.*b* = OD for blank.*c* = OD for experimental sample.

The standard curve was then generated from the concentrations of serial standard samples and their M86 inhibition rate using the method of fitting hyperbola, and plotting the concentration-M86 inhibition rate calibration curve. The following fitting equation was used:

2 $${\rm{Y}}={{\rm{Ae}}}^{(-{\rm{x}}/{\rm{t}})}+{{\rm{y}}}_{0}$$

whereY = inhibition rate of M86 binding (%).x = the concentration of standard samples (mg/mL, or µg/mL or ng/mL).A, e, t, y_0_ are constant.

Based on the formula generated from the standard curve, the concentration of each sample with the same M86 inhibition rate as the standard was calibrated, and the amount of Gal antigens was calculated relative to the standard sample (Gal-BSA).

The concentration of test sample at 50% inhibition rate (IC_50_) can be obtained by comparing with the IC_50_ of standard sample (Gal-BSA mixture), and they should contain same amount of Gal antigens at IC_50_ because they were determined under the same test system. However, only the IC_50_ of the test sample can be obtained as it contains higher levels of Gal antigens, and it would not provide an accurate calculation of Gal epitopes if used as a relative quantitative method. IC_50_ for the most decellular animal tissue-derived biomaterials that contain very low levels of remnant Gal antigens could not be obtained. In this case, the quantification should be performed using a calibration curve.

In our preliminary experiments on protein quantification, we confirmed that the mass weight of Galα1-3gal-BSA (Gal-BSA) was approximately equal to BSA alone. Relative molecular mass of BSA is 66.33 kDa, and the number of molecules is 9.08 × 10^18^/g. According to the product information, each Galα1-3gal-BSA molecule contains about 20 Gal epitopes, so the number of Gal epitopes in Galα1-3gal-BSA is 1.82 × 10^20^/g. The Gal epitopes of test sample can then be calculated relative to the standard sample (Gal-BSA).

In order to detect the Gal antigen in this system, it is important to maintain the Gal antigen in the test sample within the range of standard curve. It is necessary to detect the LDL of the test system because the decellular animal tissue-derived biomaterials contain very low levels of remnant Gal. Based on the LDL, the undetectable levels of Gal antigen in the test samples can be justified.

### Statistical analysis

Data are expressed as mean ± SD. Differences were considered significant when p < 0.05. Statisticaly significant differences between the groups were determined by one-way analysis of variance.

## Data Availability

The all of data are available.
